# Correction: Resistance profile of the HIV-1 maturation inhibitor GSK3532795 in vitro and in a clinical study

**DOI:** 10.1371/journal.pone.0224976

**Published:** 2019-11-05

**Authors:** The PLOS ONE Staff

## Notice of Republication

Incorrect versions of Figs 1–5 were published in error. The publisher apologizes for this error. This article was republished on October 24, 2019 to correct for this error. Please download this article again to view the correct version.
