# Supplementary material for: Resistance profile of the HIV-1 maturation inhibitor GSK3532795 in vitro and in a clinical study
Source: PLoS One. 2019 Oct 17;14(10):e0224076. doi: 10.1371/journal.pone.0224076 (PMC6797179; doi:10.1371/journal.pone.0224076)
Supplement: S1 Table — (PDF) [file pone.0224076.s001.pdf]

S1 Table: Amino Acid Substitutions Selected in Gag During Independent Sequential Passage Experiments in the Presence of Increasing Concentrations of GSK35323795

				Gag region[a,b]					
				Capsid				Capsid/SP1	
**Start Virus[a]**	**Start [drug], xEC_{50}**	**Expt/ culture #**	**P# (Days[c])**	**218**	**219**	**326**	**332**	**362**	**364**
V362I/ V370A	1	3/1,2.3	P11		H219Q (2/3)				
V362I	1	3/1,2.3	P11		H219Q		T332T/N (1/3)		
T332S/ V362I	1	3/1,2.3	P11		H219Q (2/3)				
T332S	1	3/1	P11		H219Q			V362V/I	
		3/2			H219Q				A364V
		3/3			H219P				A364V
R286K/ V370A	1	3/1	P11		H219Q			V362I	
		3/2		V218V/A		A326T		V362I	
		3/3				A326T		V362I	
R286K	1	3/2	P11		H219Q			V362V/I	A364A/V
		3/1,3			H219Q				A364V
ΔV370	1	3/1,2.3	P11		H219Q	A326A/T			
ΔT371	1	3/1,2.3	P11		H219Q				A364A/V

[a]WT virus was $NL_{4\text{-}3}$RepRluc Gag P373S, variant viruses were $NL_{4\text{-}3}$RepRluc Gag P373S with the indicated additional changes in Gag. Infections were initiated at a multiplicity of infection of 0.005; [b] sequence changes were observed in Matrix, SP2/P6 or protease genes; [c]Number of days in culture.
EC_{50}, 50% effective concentration; P#, passage number; SP1, spacer peptide 1; WT, wild-type
