# Supplementary material for: Resistance profile of the HIV-1 maturation inhibitor GSK3532795 in vitro and in a clinical study
Source: PLoS One. 2019 Oct 17;14(10):e0224076. doi: 10.1371/journal.pone.0224076 (PMC6797179; doi:10.1371/journal.pone.0224076)
Supplement: S2 Table — (PDF) [file pone.0224076.s002.pdf]

S2 Table: Sequences of key amino acids in Gag in subjects treated with 5-120 mg GSK3532795[a]

Dose	#	Log10 HIV-1 RNA Maximum Decline	IC50 FOLD CHANGE	*DAY*	V362	A364	Q369	V370	T371
5	6	-0.22	387.44	*-1*	V362I	A364	Q369	V370	T371
5	14	-0.48	1.62	*-1*	V362	A364	Q369	V370	T371
5	21	-0.23	2.98	*-1*	V362I	A364	Q369	V370	T371
5	23	-0.32	0.68	*-1*	V362	A364	Q369	V370	T371
5	36	-0.64	2.68	*-1*	V362I/V	A364	Q369H/Q	V370A/V	T371N
5	47	-0.7	0.9	*-1*	V362	A364	Q369	V370	T371
5	58	-0.78	1.03	*-1*	V362I/V	A364	Q369	V370	T371
5	59	-0.52	20.06	*-1*	V362I/V	A364	Q369	V370A	T371Q
10	17	-1.31	2.52	*-1*	V362	A364	Q369	V370M	T371
10	17	-1.31	1.57	*10*	V362I/V	A364	Q369	V370M	T371
10	17	-1.31	10.01	*14*	V362	A364	Q369	V370M	T371
10	19	-0.64	1.28	*-1*	V362	A364	Q369	V370	T371-
10	19	-0.64	1.07	*10*	V362	A364	Q369	V370	T371-
10	19	-0.64	1.27	*14*	V362	A364	Q369	V370	T371-
10	20	-0.91	17.52	*-1*	V362	A364	Q369	V370M	T371
10	20	-0.91	NR[b]	*10*					
10	20	-0.91	NR	*14*					
10	25	-0.97	41.05	*-1*	V362I/V	A364	Q369	V370	T371
10	25	-0.97	423.23	*10*	V362I	A364	Q369	V370	T371
10	25	-0.97	>617	*14*	V362I	A364	Q369	V370	T371
10	34	-1.76	0.63	*-1*	V362	A364	Q369	V370	T371
10	34	-1.76	42.24	*10*	V362	A364A/V	Q369	V370	T371
10	34	-1.76	>667	*14*	V362I/V	A364A/V	Q369	V370	T371
10	43	-0.74	>633	*-1*	V362I	A364	Q369	V370	T371N
10	43	-0.74	>667	*10*	V362I	A364	Q369	V370	ΔT371

10	43	-0.74	>667	*14*	V362I	A364	Q369	V370	T371N
10	48	-1.05	0.99	*-1*	V362	A364	Q369	V370	T371
10	48	-1.05	NR	*10*					
10	48	-1.05	2.19	*14*	V362	A364	Q369	V370	T371
10	55	-0.98	>633	*-1*	V362I	A364	Q369	V370A	T371
10	55	-0.98	453.52	*10*	V362I	A364	Q369	V370A	T371
10	55	-0.98	>667	*14*	V362I	A364	Q369	V370A	T371
20	2	-1	398.75	*-1*	V362	A364	Q369	V370	T371
20	2	-1	648.95	*10*	V362	A364	Q369	V370	T371
20	2	-1	>667	*14*	V362	A364	Q369	V370L/V	T371
20	13	-1.68	0.48	*-1*	V362	A364	Q369	V370	T371
20	13	-1.68	0.52	*10*	V362	A364	Q369	V370I/V	T371
20	13	-1.68	1.01	*14*	V362	A364A/V	Q369	V370	T371
20	18	-0.89	NR	*-1*					
20	18	-0.89	NR	*10*					
20	18	-0.89	NR	*14*					
20	26	-1.23	1.66	*-1*	V362	A364	Q369	V370A	T371N
20	26	-1.23	>667	*10*	V362I/V	A364A/V	Q369	V370A	T371N
20	26	-1.23	>667	*14*	V362I/V	A364A/V	Q369	V370A	T371N
20	31	-0.13	>633	*-1*	V362	A364	Q369	V370A/V	T371
20	31	-0.13	63.86	*10*	V362	A364	Q369	V370A	T371
20	31	-0.13	NR	*14*					
20	42	-0.63	>633	*-1*	V362I	A364	Q369	V370I	T371
20	42	-0.63	>602	*10*	V362I	A364	Q369	V370I	T371
20	42	-0.63	>667	*14*	V362I	A364	Q369	V370I	T371
20	52	-2.12	0.48	*-1*	V362	A364	Q369	V370M	T371
20	52	-2.12	106	*10*	V362	A364A/V	Q369	V370M	T371
20	52	-2.12	227.4	*14*	V362	A364A/V	Q369	V370M	T371
20	57	-1.66	0.48	*-1*	V362	A364	Q369	V370	T371
20	57	-1.66	0.51	*10*	V362	A364	Q369	V370	T371

20	57	-1.66	0.41	*14*	V362	A364	Q369	V370	T371
40	1	-1.09	0.39	*-1*	V362	A364	Q369	V370	T371
40	1	-1.09	3.79	*10*	V362I/V	A364A/V	Q369	V370	T371
40	1	-1.09	>667	*14*	V362	A364A/V	Q369	V370	T371
40	10	-1.77	103.85	*-1*	V362	A364	Q369	V370M	T371
40	10	-1.77	267.89	*10*	V362	A364	Q369	V370M	T371
40	10	-1.77	>667	*14*	V362I/V	A364A/V	Q369	V370M	T371X
40	11	-1.54	NR	*-1*					
40	11	-1.54	NR	*10*					
40	11	-1.54	NR	*14*					
40	22	-0.93	1.63	*-1*	V362	A364	Q369	V370M	T371
40	22	-0.93	20.28	*10*	V362	A364	Q369H/Q	V370M	T371
40	22	-0.93	23.47	*14*	V362I/V	A364	Q369H/Q	V370M	T371
40	32	-1.88	0.4	*-1*	V362	A364	Q369	V370	T371
40	32	-1.88	0.41	*10*	V362	A364	Q369	V370	T371
40	32	-1.88	NR	*14*					
40	38	-1.71	68.53	*-1*	V362	A364	Q369	V370A/V	T371
40	38	-1.71	495.75	*10*	V362	A364	Q369	V370A	T371
40	38	-1.71	>667	*14*	V362I/V	A364	Q369	V370A	T371
40	40	-1.82	0.68	*-1*	V362	A364	Q369	V370	T371
40	40	-1.82	1.79	*10*	V362	A364	Q369	V370	T371
40	40	-1.82	0.59	*14*	V362	A364	Q369	V370	T371
40	44	-1.69	1.14	*-1*	V362	A364	Q369	V370	T371
40	44	-1.69	132.05	*10*	V362	A364	Q369	V370M	T371
40	44	-1.69	0.48	*14*	V362	A364	Q369	V370	T371
80	82	-1.32	1.63	*-1*	V362	A364	Q369	V370	T371Q
80	82	-1.32	2.06	*10*	V362	A364	Q369	V370	T371Q
80	82	-1.32	0.74	*14*	V362	A364	Q369	V370	T371Q
80	85	-1.5	3.13	*-1*	V362	A364	Q369	V370	T371
80	85	-1.5	145.94	*10*	V362I/V	A364A/V	Q369	V370	T371

80	85	-1.5	>581	*14*	V362I/V	A364A/V	Q369	V370	T371
80	86	-1.04	2.03	*-1*	V362	A364	Q369	V370M	T371
80	86	-1.04	2.26	*10*	V362	A364	Q369	V370M	T371
80	86	-1.04	>581	*14*	V362I/V	A364	Q369	V370M	T371
80	88	-1.6	0.41	*-1*	V362	A364	Q369	V370	T371
80	88	-1.6	0.51	*10*	V362	A364	Q369	V370	T371
80	88	-1.6	1.38	*14*	V362	A364A/V	Q369	V370	T371
80	90	-1.82	0.38	*-1*	V362	A364	Q369	V370I/V	T371
80	90	-1.82	0.52	*10*	V362	A364	Q369	V370I/V	T371
80	90	-1.82	96.42	*14*	V362I/V	A364A/V	Q369	V370I/V	T371
80	98	-1.73	0.95	*-1*	V362	A364	Q369	V370	T371
80	98	-1.73	0.94	*10*	V362	A364A/V	Q369	V370	T371
80	98	-1.73	>581	*14*	V362	A364A/V	Q369	V370	T371
80	99	-1.72	0.86	*-1*	V362	A364	Q369	V370	T371
80	99	-1.72	2.4	*10*	V362	A364	Q369	V370	T371
80	99	-1.72	NR	*14*					
80	102	-1.4	0.59	*-1*	V362	A364	Q369	V370	T371
80	102	-1.4	0.7	*10*	V362	A364	Q369	V370	T371
80	102	-1.4	0.85	*14*	V362	A364A/V	Q369	V370	T371
120	6	-2.02	4.42	*-1*	V362	A364	Q369	V370-	T371
120	6	-2.02	4.39	*10*	V362	A364	Q369	V370-	T371
120	6	-2.02	17.99	*14*	V362	A364	Q369	V370-	T371
120	106	-1.98	36.89	*-1*	V362	A364	Q369	V370M	T371
120	106	-1.98	4.01	*10*	V362	A364	Q369	V370M	T371
120	106	-1.98	337.2	*14*	V362	A364A/V	Q369	V370M	T371
120	109	-0.83	2.95	*-1*	V362I/V	A364	Q369	V370	T371
120	109	-0.83	407.06	*10*	V362I	A364	Q369	V370	T371
120	109	-0.83	214.11	*14*	V362I	A364A/V	Q369	V370	T371
120	110	-2.07	0.82	*-1*	V362	A364	Q369	V370A/V	T371
120	110	-2.07	5.2	*10*	V362	A364	Q369	V370X	T371X

120	110	-2.07	0.89	*14*	V362	A364	Q369	V370	T371
120	111	-1.57	3.85	*-1*	V362	A364	Q369	V370	T371
120	111	-1.57	15.83	*10*	V362	A364	Q369	V370A/V	T371
120	111	-1.57	>704	*14*	V362	A364	Q369	V370A/V	T371
120	112	-1.74	1.58	*-1*	V362	A364	Q369	V370	T371
120	112	-1.74	2.17	*10*	V362	A364	Q369	V370	T371
120	112	-1.74	27.01	*14*	V362	A364A/V	Q369	V370	T371
120	115	-1.81	0.7	*-1*	V362	A364	Q369	V370	T371
120	115	-1.81	1.56	*10*	V362	A364	Q369	V370	T371
120	115	-1.81	>704	*14*	V362	A364A/V	Q369	V370	T371
120	116	-1.42	-1.42	*-1*	V362	A364	Q369	V370	T371
120	116	-1.42	0.42	*10*	V362	A364	Q369	V370	T371
120	116	-1.42	0.56	*14*	V362	A364A/V	Q369	V370	T371
PBO[b]	83	0.08	2.15	*-1*	V362	A364	Q369	V370	T371
PBO	83	0.08	1.35	*10*	V362	A364	Q369	V370	T371
PBO	83	0.08	1.64	*14*	V362	A364	Q369	V370	T371
PBO	91	-0.33	8.44	*-1*	V362	A364	Q369	V370L	T371
PBO	91	-0.33	20.72	*10*	V362	A364	Q369	V370L	T371
PBO	91	-0.33	14.41	*14*	V362	A364	Q369	V370L	T371
PBO	105	-0.6	183.72	*-1*	V362	A364	Q369	V370L	ΔT371
PBO	105	-0.6	133.86	*10*	V362	A364	Q369	V370L	ΔT371
PBO	105	-0.6	103.46	*14*	V362	A364	Q369	V370L	ΔT371
PBO	126	-1.05	1.08	*-1*	V362	A364	Q369	V370X	T371X
PBO	126	-1.05	1.65	*10*	V362	A364	Q369	V370X	T371X
PBO	126	-1.05	1.55	*14*	V362	A364	Q369	V370X	T371X

[a]: Subjects treated on Day 1 with the indicated dose of GSK3532795 for 10 days. Sequences of Gag were obtained at Days ‘1, 10 and 14; Samples at Day 10 and 14 for the 5 mg cohort were not analyzed due to lack of an antiviral effect; Amino acids found at positions 363, 364, 369-371 are shown; green cells indicate same sequence as consensus; purple cell indicates a non-consensus sequence or mixture; [b]: NR = Nonreportable; PBO = placebo dosed.
