# Supplementary material for: Resistance profile of the HIV-1 maturation inhibitor GSK3532795 in vitro and in a clinical study
Source: PLoS One. 2019 Oct 17;14(10):e0224076. doi: 10.1371/journal.pone.0224076 (PMC6797179; doi:10.1371/journal.pone.0224076)
Supplement: Attachment — Submitted filename: Reviewer_Comments.docx [file pone.0224076.s003.docx]

Reviewer comments:

GSK3532795 (formerly BMS955176) is a second-generation maturation inhibitor (MI) that has advanced to Phase 2b clinical trial for treatment of HIV-1 infection but discontinued from further development. In this study, resistance development to GSK3532795 was evaluated through *in vitro* methods by using various strategies. The reduced susceptibility to GSK3532795 mapped specifically to amino acids near the capsid/ spacer peptide 1 (SP1) junction, the cleavage of which is blocked by MIs. Two key substitutions, A364V or V362I, were selected, along with few other secondary substitutions. These mutations were similar to the resistance profile of BVM. Mutations acquired in *in vitro* experiments were then correlated with information obtained in a Phase 2a proof-of-concept study in HIV-1 infected participants. In the Phase 2a study, a subset of these substitutions was also observed at baseline and some were selected following GSK35323795 treatment in HIV-1-infected participants. All the emergent substitutions observed in this study were similar to a subset observed during *in vitro* selections with this compound. This study suggested the relevance of *in vitro* selection studies for evaluation of the resistance profile of maturation inhibitors. Furthermore, in this study, they structurally modelled the compound with immature capsid hexamer which suggested possible mechanism of action and interaction of MIs and capsid protein was similar for Bevirimat as well as GSK3532795. This is a well carried out study. I have the following suggestions:

**Comments:**

1. Abstract: Line no. 36: According to the present study, H219Q increased viral replication capacity and reduced susceptibility of poorly growing viruses “only in *in vitro s*tudies”. (Reference required)
2. Introduction: Line no. 57: Mention the studies done to identify other BVM resistant mutations (Reference required).
3. Introduction: Line no. 58: Add another reference Waki *et.al*, 2012, as they have identified more resistant mutants in presence of the same maturation inhibitor.
4. Introduction: Line no. 61: Reference no. 10 is not relevant in this case.
5. Materials and methods: Line no. 94-100: Please mention about the no drug control used in the study.
6. Materials and methods: Line no. 166: Please give full form of QD.
7. Materials and methods: Line no. 166: Only part A of AI68002 clinical study was used in this study. Hence there is no need to mention about part C. If it has to be mentioned here, please justify why part C was not used in the present study.
8. Results: Page no. 10: Along with the table 1, a pictorial representation of the sequence of CA-SP1 region which shows locations of mutated amino acids will help in better understanding of mutation pattern.
9. Results: Line no. 224: Reference is missing.
10. Results: Page no. 13: Along with the table 2, a pictorial representation of the sequence of CA-SP1 region which shows locations of mutated amino acids will help in better understanding of mutation pattern.
11. Results: Line no: 240-247: The paragraph reads rephrasing…
12. Results: Line no. 258: please explain the meaning of square?
13. Results: Line no 258: In the WT virus at amino acid position 371, there is T instead of V as mentioned. Please explain
14. Results: Line no. 264: Same as comment no. 13.
15. Results: Page no. 15: Along with the table 3, a pictorial representation of the sequence of CA-SP1 region which shows locations of mutated amino acids will help in better understanding of mutation pattern.
16. Results: Line no 275: Please add reference of Adamson *et.al*, 2006 also, as this work is also relevant.
17. Results: Line no. 287: Please add reference for the statistics given.
18. Results: Line no 289-290: Please rewrite the sentence.
19. Results: Line no. 303-304: The sentence is not clear.
20. Results: Line no. 333-339: It is clearly mentioned that 8 samples contains two or more Gag polymorphisms. But from the sentence “Analysis of the genotypes showed that 5 participants had polymorphisms at both positions 370 and 371 (one had undetermined amino acids at 370 and 371), two participants contained polymorphisms at positions 362 and 370, one participant had polymorphisms at 362, 370 and 371 and one participant contained polymorphisms at positions 362 and 369-371.”, the total patients become 9. Please check it carefully.
